# Synthesis of bio-based N-containing compounds from biomass

**DOI:** 10.1093/nsr/nwaf450

**Published:** 2025-10-21

**Authors:** Jianwei Ji, Wenqing Zhu, Bo Zhang, Julian Skagfjörd Reinhold, Aiqin Wang, Tao Zhang

**Affiliations:** Shaanxi Key Laboratory of Catalysis, School of Chemistry and Environment Science, Shaanxi University of Technology, Hanzhong 723001, China; CAS Key Laboratory of Science and Technology on Applied Catalysis, Dalian Institute of Chemical Physics, Chinese Academy of Sciences, Dalian 116023, China; Molecular Catalysis, Catalysis Research Center and Department of Chemistry, School of Natural Sciences, Technical University of Munich, Garching bei München D-85748, Germany; CAS Key Laboratory of Science and Technology on Applied Catalysis, Dalian Institute of Chemical Physics, Chinese Academy of Sciences, Dalian 116023, China; University of Chinese Academy of Sciences, Beijing 100049, China; CAS Key Laboratory of Science and Technology on Applied Catalysis, Dalian Institute of Chemical Physics, Chinese Academy of Sciences, Dalian 116023, China; Molecular Catalysis, Catalysis Research Center and Department of Chemistry, School of Natural Sciences, Technical University of Munich, Garching bei München D-85748, Germany; CAS Key Laboratory of Science and Technology on Applied Catalysis, Dalian Institute of Chemical Physics, Chinese Academy of Sciences, Dalian 116023, China; University of Chinese Academy of Sciences, Beijing 100049, China; CAS Key Laboratory of Science and Technology on Applied Catalysis, Dalian Institute of Chemical Physics, Chinese Academy of Sciences, Dalian 116023, China; University of Chinese Academy of Sciences, Beijing 100049, China

**Keywords:** biomass conversion, N-containing compounds, lignin

## Abstract

Lignocellulose offers a high potential for replacing fossil fuels. Its primary components—cellulose, hemicellulose, and lignin—can be catalytically transformed into value-added chemicals. The involvement of nitrogen in the degradation of lignocellulose to produce bio-based nitrogen (N)-containing compounds is emerging as a key research focus. This research area is very important because it not only transcends the conventional limitations of C/H/O-containing compounds production but also offers substantial theoretical and practical insights into the rational utilization of lignocellulosic feedstocks. Therefore, with this review we provide insights into accomplishments for the transformation of major components of lignocellulosic biomass, including polysaccharide-derived platform compounds, lignin model compounds, lignin and lignocellulose feedstocks, into value-added N-containing compounds. The focus is on analyzing catalytic systems and reaction mechanisms involving C–O/C–C bond cleavage, as well as C–N bond formation. An outlook is offered to outline the key challenges and future perspectives in this rapidly growing field.

## Teaser

This review summarizes the recent advances in synthesizing bio-based N-containing compounds from lignocellulosic biomass, offering sustainable strategies for their application in pharmaceuticals and materials science.

## INTRODUCTION

**Figure 1.**
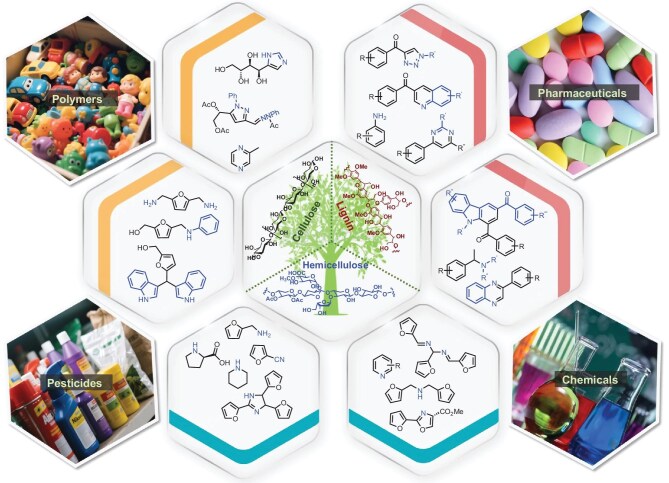
The structure of lignocellulose and production of bio-based N-containing compounds.

Among various renewable energy sources, lignocellulosic biomass resources exhibit characteristics including wide availability, abundant reserves, and carbon-neutrality, therefore representing an effective approach to address modern energy and environmental challenges by substituting fossil fuels [1]. Lignocellulose primarily consists of cellulose, hemicellulose, and lignin (Fig. 1). Structurally and chemically, cellulose is a high-molecular-weight polymer composed of glucose units linked by β-1,4-glycosidic bonds, characterized by dense packing, high crystallinity, and extensive intra- and intermolecular hydrogen bonding [2]. Hemicellulose is a heterogeneous polysaccharide composed of diverse monosaccharides, including pentoses and hexoses, whereas lignin—formed through C–O and C–C linkages of coniferyl, sinapyl, and *p*-coumaryl alcohol units—represents the primary renewable source of aromatic compounds in nature [3]. Based on the composition and characteristics of lignocellulose, catalytic conversion technologies are employed to produce value-added chemicals and fuels, achieving the dual benefits of environmental pollution reduction and economic enhancement.

Nitrogen (N)-containing compounds have extensive applications in pharmaceuticals, agrochemicals, polymers, and functional materials [4]. Current synthetic methods predominantly rely on fossil resources, non-renewable feedstocks suffering from harsh reaction conditions and excessive waste generation, which pose significant environmental challenges. Developing new strategies for producing N-containing compounds from renewable biomass enables reducing fossil fuel dependence and environmental pressure, and has thus become a hot topic in biomass conversion research. This strategy primarily involves selective transforming of the C–O/C–C bond into a C–N bond. The main method of C–N bond construction is the reductive amination of aldehydes and ketones. This mechanism allows for the selective conversion of carbonyl groups to amino groups in the presence of H-sources, even at low temperatures [5]. A particularly important method for C–N bond formation is the hydrogen auto-transfer process, involving either borrowing hydrogen or self-supplying active hydrogen. Catalytic conversion of alcohol groups into the corresponding amines proceeds without H_2_ consumption. These reactions are typically achieved at high temperatures (>150°C) owing to the formation of a carbonyl group through dehydrogenation of alcohol substrates, which is then attacked by nucleophilic nitrogen sources [6]. An additional promising strategy is hydroamination, where an amine molecule is added directly to an unsaturated (double or triple) C–C bond. However, catalytic hydroamination remains limited in scope. Kinetic challenges arise from high activation barriers in coupling two substrates and catalyst deactivation via amine coordination. Additionally, the reaction is often thermodynamically unfavorable [7]. Considering that renewable resources and biomass-derived platform molecules contain high quantities of hydroxyl groups, therefore, dehydrogenation of alcohol groups to aldehydes or ketones followed by reductive amination could be a preferred strategy.

Based on progress on the synthesis of bio-based N-containing compounds from lignocellulosic biomass, several reviews have addressed this topic. Sels’ group reviewed amination reactions of biomass-derived polyols and lignin model compounds for nitrogen heterocycle synthesis [5]. Li *et al.* [8] summarized heteroatom (N, Si, I, Li)-assisted lignin depolymerization strategies yielding aromatic amines, amides, siloxanes, and lithium phenolate derivatives. Froidevaux *et al.* [9] documented bio-based amine synthesis from chitin, lysine, and vegetable oils for polymer applications. Most reviews in this field emphasize a particular reaction or address only single-component-lignin depolymerization. In recent years, numerous promising catalytic methods have emerged, often affording unexpected product structures. Highlighting achievements in the production of bio-based N-containing compounds from biomass conversion, a systematic summary of N-containing compounds from cellulose, hemicellulose, and lignin has not yet been covered, particularly with the newly emerging nitrogen-assisted lignin cleavage to produce functionalized N-containing aromatics. Therefore, the core of this review summarizes the recent advances in targeted synthesis of bio-based N-containing compounds from these three components and their derived platform molecules. Attention is focused on both the reaction route and mechanism of transforming lignocellulose into N-containing compounds, giving their technological significance and market potential. Moreover, challenges and opportunities for N-containing compounds from biomass conversion are discussed.

## SYNTHESIS OF BIO-BASED N-CONTAINING COMPOUNDS FROM POLYSACCHARIDES-DERIVED PLATFORM COMPOUNDS

### Monosaccharides

**Figure 2.**
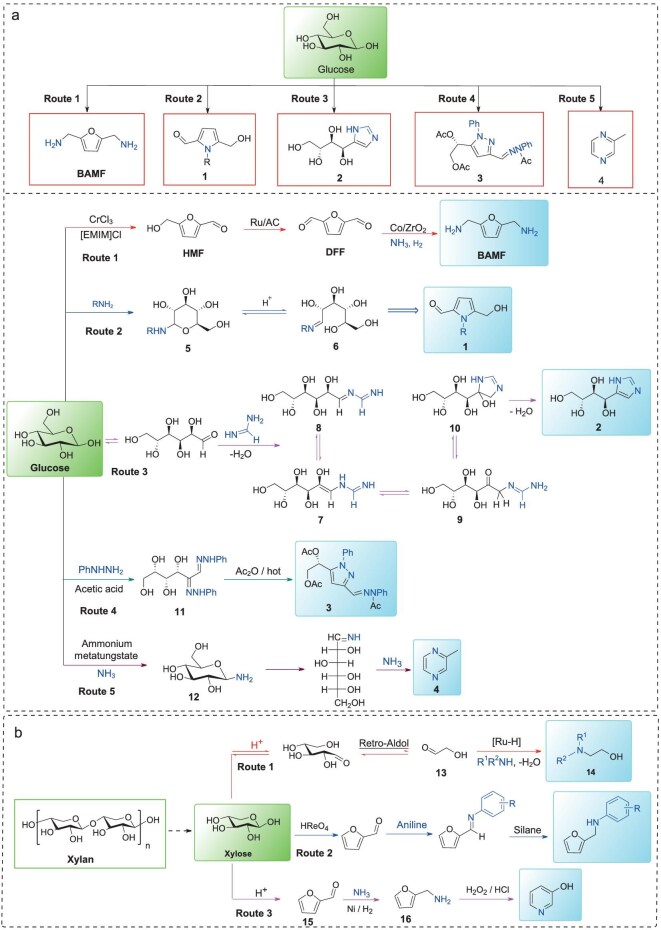
The synthesis of various N-containing compounds from (a) glucose and (b) xylose conversion.

Glucose, a key monosaccharide, can be derived from plant biomass through the hydrolysis of cellulose [10]. The transformation of glucose is crucial across various industries, including biofuels, food production, and chemical synthesis. It serves as a key precursor for multiple valuable compounds including 5-HMF, sorbitol, gluconic acid, and glucaric acid [10]. Recent progress has been made in the nitrogen-functionalization of glucose, leading to various N-containing compounds. Among them, bis(aminomethyl)furan (BAMF) represents one of the most promising primary diamines for synthesizing novel polymers with unique functionalities [11]. Our group found that the Co/ZrO_2_-catalyzed reductive amination reaction of 2,5-diformylfuran (DFF) led to BAMF through the addition of *n*-butylamine that aimed to suppress DFF self-polymerization under NH_3_ and H_2_ atmosphere [12]. Employing glucose as a substrate, this catalytic system leads to a 35% yield of BAMF based on glucose through multiple reaction steps (Fig. 2a, Route 1). In contrast to multi-step pathways, a direct conversion of D-glucose to N-substituted pyrrole-2-carbaldehydes (**1**) has been developed via its reaction with primary amines and oxalic acid in dimethyl sulfoxide (DMSO) at 90°C (Fig. 2a, Route 2) [13]. It is found that the reaction of D-glucose with aliphatic amines (*n*-butyl-, allyl-, propargyl-, 3-hydroxypropyl-, and phenethylamines) afforded the corresponding N-substituted 5-(hydroxymethyl)pyrrole-2-carbaldehydes in yields ranging from 21% to 48%. The reaction initiates with N-glycosylation of the amine to form compound **5**. In the presence of H^+^, the six-membered ring undergoes ring-opening to generate imine **6**. Subsequent protonation and dehydration of the hydroxyl group, followed by cyclization and elimination steps, ultimately lead to aromatization of the pyrrole ring to obtain product **1**.

Imidazoles are abundant in bioactive natural products and display diverse pharmacological activities [14], and can be obtained from d-glucose. For example, Brust and Cuny [15] developed a method for the production of imidazole compounds from glucose under ambient pressure (Fig. 2a, Route 3). A mixture of ammonium carbonate, methylformamidine acetate, and ammonium acetate was heated, during which ammonium carbonate decomposed to release ammonia, water, and carbon dioxide. Under these conditions, the generated amidine underwent carbonyl condensation with reducing sugars to form imine intermediate **7**. Subsequent Amadori rearrangement of intermediate **7** yielded compounds **8** and **9**, followed by cyclization of **9** to produce compound **10**. Finally, dehydration-aromatization of **10** afforded imidazole **2** with a 50% yield. This system is applicable to reducing sugars, including fructose, lactose, and maltose, enabling cost-effective imidazole synthesis from renewable sugars.

Aiming to obtain various N-containing compounds, the synthesis of pyrazole derivatives (**3**) via the reaction of glucose with phenylhydrazine in the presence of acetic anhydride was developed by EI Khadem *et al.* (Fig. 2a, Route 4) [16,17]. Alternatively, under microwave irradiation, compound **11** was converted to pyrazole derivative **3** within 9 min in an 86% yield [18]. Compared to conventional heating, microwave-assisted reactions significantly reduce the reaction time. Notably, this method also enables the synthesis of pyrazoles from galactose-, arabinose-, and xylose-derived azaketones with yields of up to 96%. Moreover, besides organic amines, NH_3_ can be used as an N-source for the synthesis of N-containing compounds from D-glucose conversion. In the presence of ammonium metatungstate, the one-pot conversion of glucose to 2-methylpyrazine **4** in 25.6% yield is achieved (Fig. 2a. Route 5) [19]. The mechanism proceeds via two key pathways: (i) a retro-aldol reaction of **12**, yielding a C2 fragment, and (ii) the isomerization of **12** to fructosylamine, followed by a retro-aldol reaction, yielding a C3 fragment. These fragments subsequently combine to form 2-methylpyrazine. The tungsten catalyst facilitates the cleavage of glucose and its subsequent cyclization to form pyrazine rings, wherein the [HW_2_O_7_]^−^ and [W_4_O_13_]^2−^ likely act as the active species responsible for the transformation. This catalytic system is applicable to a range of other monosaccharides as well as certain disaccharides.

Xylose, as a major component of hemicellulose, is an important building block for various products, including furfural, furfuryl alcohol, xylitol, levulinic acid (LA), and levulinic ester [2]. N-introduced transformation of xylose has also been extensively investigated in Fig. 2b. For instance, a study by Jia *et al.* employed a ruthenium catalyst coordinated with organophosphine ligands as the catalyst to synthesize β-amino alcohols from biomass-derived carbohydrates (Fig. 2b, Route 1) [20]. Under acidic conditions, the Ru-complex catalyst selectively cleaved C5/C6 sugars to generate C2 product glycolaldehyde (**13**), which condensed with amines to form imine intermediates. Subsequent hydrogenation yielded β-amino alcohols (**14**). This halogen-free method demonstrated high chemoselectivity and functional group tolerance, enabling efficient synthesis of β-amino alcohols from diverse substituted aromatic amines. Caetano and Fernandes developed a one-pot multi-step strategy using HReO_4_ as a catalyst to convert xylose and xylan into furfuryl secondary and tertiary amines with high chemoselectivity (Fig. 2b, Route 2) [21]. This approach established a novel route for the targeted synthesis of furfurylamines from pentoses. Alternatively, catalytic systems employing non-precious metals such as nickel can also be applied for the conversion of xylose into N-containing compounds. For instance, the Lichtenthaler group developed a simple and scalable three-step reaction pathway to synthesize 3-pyridinol from D-xylose (Fig. 2b, Route 3) [22]. The process involves acid-catalyzed hydrolysis/dehydration to furfural **15**, reductive amination to furfurylamine **16**, and subsequent oxidation mediated by 30% H_2_O_2_ in 3 mol·L^−1^ HCl.

### 5-Hydroxymethylfurfural (5-HMF)

**Figure 3.**
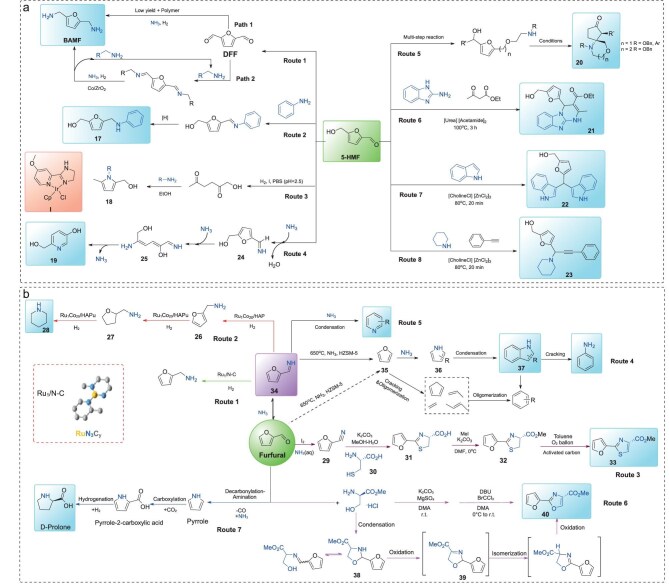
The synthesis of N-containing compounds from (a) 5-HMF and (b) furfural conversion.

5-Hydroxymethylfurfural (5-HMF), recognized by the U.S. Department of Energy as one of the 12 most promising bio-based platform compounds [23], is a critical biomass-derived building block. Interest in using 5-HMF as a starting material for the production of value-added chemicals is currently increasing. For instance, 5-HMF can be selectively oxidized to 2,5-furandicarbaldehyde (DFF) and 2,5-furandicarboxylic acid (FDCA), which serve as key precursors for synthesis of pharmaceuticals and novel polymer materials. The selective hydrogenation of 5-HMF to 2,5-bishydroxymethyltetrahydrofuran (BHMTHF), an important precursor, is crucial for biomass refining [24]. Beyond oxidation and hydrogenation reactions, amination of 5-HMF has attracted significant attention owing to BAMF production. However, current synthetic approaches for BAMF yield unsatisfactory results (46.7%–85.9%) due to the facile polymerization between diamines and dialdehydes [25]. To enhance BAMF yield, an alternative strategy introduced alkylamines into the reaction system to suppress DFF polymerization, achieving a 95% yield of BAMF through the Co/ZrO_2_-catalyzed reductive amination reaction (Fig. 3a, Route 1) [12]. The reaction mechanism is as follows: the excess organic amine reacts rapidly with DFF to form bis N-alkylimine, which inhibits the occurrence of side reactions. Subsequently, in the presence of NH_3_, bis N-alkylimine undergoes a reversible imine exchange reaction, slowly forming a stable primary NH-imine with low concentration. Once the primary NH-imine is formed, it is rapidly hydrogenated by the Co/ZrO_2_ catalyst to produce the target product BAMF. Apart from BAMF production, current efforts focus on developing multiple successful reactions to obtain various types of N-containing compounds **17–23** from 5-HMF conversion. For example, Zhang and co-workers [26] employed various ruthenium-based complexes as catalysts for the reductive amination of 5-HMF with aniline (Fig. 3a, Route 2). Under the reaction conditions of 60°C and 1.2 MPa H_2_ pressure, an exceptional 98% yield of N-phenyl-5-(hydroxymethyl)-2-furanamide (**17**) was obtained within 5 h. Mechanistic studies suggest that the condensation between 5-HMF and aniline leads to the corresponding imine intermediate, followed by its hydrogenation to yield the final amine product. Furthermore, an Ir-based homogeneous complex was developed by Wozniak and co-workers to convert 5-HMF to N-substituted 2-hydroxymethyl-5-methylpyrroles **18** in 74%–99% yields (Fig. 3a, Route 3) [27]. Hereby the pathway was proposed. First, complete conversion of 5-HMF to 1-hydroxyhexane-2,5-dione (HHD) catalyzed by iridium complex **I** in phosphate-buffered saline (PBS, pH = 2.5) with ethanol as solvent at room temperature is achieved, affording 71% isolated yields. Subsequently, reaction with amines and anilines bearing both electron-withdrawing and electron-donating groups via the Paal–Knorr reaction yields product **18**. The research group also proposed an alternative strategy for the synthesis of benzenic aromatics from 5-HMF. This approach involves the catalytic oxidation of 5-HMF to a key intermediate, 2,5-dioxohexanedial (DOH), obtained in 65% overall yield. Subsequently, over acid catalysis, the selective formation of either 4-dialkylaminophenol (up to 69% yield) or 1,4-bis(dialkylamino)benzene was achieved by controlling the addition of secondary amines and the type of acid used (trifluoroacetic acid (TFA), TFA/acetic acid) [28]. In addition, the annulation of 5-HMF with reaction derivatives to form pyridin-3-ol **19** was investigated (Fig. 3a, Route 4) [29]. Under neutral pH conditions, 5-HMF initially reacts with ammonia to generate imine intermediate **24**. The introduction of a second ammonia molecule induces electronegativity-driven polarization of the C–O bond in the furan ring, leading to ring-opening of **24** to form intermediate **25**. Subsequent cyclization of **25** yields pyridine derivative **19**. Studies by Larduinat *et al.* demonstrated the synthesis of ketonic aza-spirocyclic compounds from 5-HMF via intramolecular aza-Piancatelli reactions (Fig. 3a, Route 5) [30]. Their study explored the effects of nucleophiles, aryl amines, and alkoxyamines on the reactivity, enabling the diversification of [4.5]- and [4.6]-spirocycle libraries. The intramolecular aza-Piancatelli rearrangement serves as the key step, delivering moderate to high yields with excellent dia-stereoselectivity.

Ionic liquids are often used as the solvent for biomass conversion owing to their excellent thermal stability, low vapor pressure, ability to dissolve a wide range of organic and inorganic substrates, and high electrical conductivity [31]. As an illustration, an ionic liquid (IL)-mediated strategy has been developed for the efficient conversion of carbohydrates (fructose, glucose, sucrose, and cellulose) to 5-HMF, in which ILs function as both solvent and catalyst. Notably, the as-prepared 5-HMF in deep eutectic solvents reacted with various organic amines successfully transforming it into N-containing heterocycles **21–23** (Fig. 3a, Routes 6–8) [32]. In detail, it underwent a one-pot, three-component reaction with 2-aminobenzimidazole and ethyl acetoacetate to afford **21** (Fig. 3a, Route 6). It also participated in electrophilic substitution with indoles to afford **22** (Fig. 3a, Route 7), and coupling with phenylacetylene and piperidine to yield **23** in a deep eutectic solvent of choline chloride and zinc chloride (Fig. 3a, Route 8). These transformations highlight the potential of 5-HMF as a precursor for bioactive heterocycles under benign conditions.

### Furfural

Furfural, regarded as a top added-value platform molecule for the manufacture of chemicals and fuels, is produced by acid-catalyzed treatment of agricultural waste including corn cobs or sugar cane bagasse [33]. Recent progress in converting furfural into medicinal intermediates—including pyrroles, thiazoles, pyridines, pyrazines, and indoles—via reactions with nitrogen sources has gained prominence, extending its applications in the pharmaceutical field [34,35]. Herein, this part focuses on the development of new strategies and discusses proposed mechanisms for N-containing compounds from furfural as well as their applications.

Among various furfural derivatives, furfurylamine is an important furfural-derived chemical and a key precursor for pharmaceuticals and agrochemicals [35]. For the rational design of catalysts applied in the reductive amination of furfural to produce primary amines, it is essential to achieve effective modulation of the hydrogenation activity and establish a structure–performance relationship at the atomic/molecular level. Among various catalytic strategies, single-atom catalysts (SACs) combine the merits of homogeneous catalysts (isolated active sites with high activity/selectivity) and heterogeneous catalysts (structural stability and facile separation), positioning them as bridges between heterogeneous and homogeneous catalytic systems [36]. Our group [37] used single atom Ru_1_/NC as catalyst for the conversion of furfural, affording furfurylamine in 97% yield (Fig. 3b, Route 1). Compared with nanoparticle catalyst Ru/AC, the SAC exhibited superior CO and sulfur poisoning resistance, along with remarkable catalytic activity and cycling stability (no deactivation observed after 5 cycles). The same group found that the different Ru-based single atom catalyst leads to the formation of piperidine (Fig. 3b, Route 2). Remarkably, multifunctional surface single-atom alloy Ru_1_Co_20_/HAP (hydroxyapatite, HAP) exhibits exceptional activity for one-pot conversion of furfural to piperidine, delivering the desired product in 93% yield under mild conditions [38]. Kinetic studies revealed a three-step reaction pathway: (1) amination of furfural to form furfurylamine **26**, (2) subsequent hydrogenation to tetrahydrofurfurylamine **27**, and (3) final ring rearrangement to produce piperidine **28**. The ring-opening of tetrahydrofurfurylamine was identified as the rate-determining step. Mechanistic investigations demonstrated that catalyst support promotes Ru dispersion, and that the resulting Ru–Co single-atom alloy structure is crucial for the hydrogenative rearrangement of tetrahydrofurfurylamine to piperidine.

Transition-metal free synthesis of 2-furylthiazole-4-methyl carboxylate **33** from furfural is achieved through amination, condensation, and oxidative aromatization (Fig. 3b, Route 3) [39]. The pathway involves: (1) furfural ammonolysis to afford intermediate **29**; (2) condensation with **30** under mild alkaline conditions (60°C, methanol/water) yielding 4-carboxy-2-furylthiazolidine **31**; (3) esterification with iodomethane in *N,N*-dimethylacetamide (DMF) at 0°C to give furylthiazolidine **32** (63% yield); and (4) oxidative dehydrogenation of **32** with activated carbon in toluene at 100°C to furnish the target compound **33** (97% yield). Compound **33** exhibits biofluorescence properties, rendering it applicable as a luminescent material [40].

As an important fine chemical, indole is widely used in medicine, pesticides and other fields, and can be produced from furfural through multi-step reactions [41]. In a study conducted by Zhang *et al.* [42], an indole synthesis protocol using HZSM-5 catalyst (Si/Al = 25) is developed (Fig. 3b, Route 4). The optimized conditions were a weight hourly space velocity (WHSV) of 1.0 h^−1^, reaction temperature of 650°C, and a NH_3_/furfural molar ratio of 2. This system achieved a 20.8% indole yield through gas-phase conversion. The authors revealed that both acidity and pore architecture of the HZSM-5 zeolite play a pivotal role in dictating indole formation. Specifically, the optimized acidic sites (Si/Al = 25) were effective in catalyzing the cleavage of furfural imine intermediates and subsequent cyclization of pyrrole moieties. Concurrently, the microporous framework, featuring precisely tailored 0.5-nm channels, exhibited exceptional molecular-sieving capabilities. A plausible reaction pathway by quantum chemical calculations and catalytic testing showed that furfural initially reacts with ammonia to form a furfural-imine intermediate **34**, which subsequently undergoes cleavage to yield furan **35**. Furthermore, the newly formed furan then engages in ammonolysis, generating pyrrole along with various alkylpyrrole derivatives **36**. These pyrrolic compounds participate in either ring-opening transformations or Diels–Alder condensations—both self-condensation between pyrrole molecules and cross-reactions with furan ultimately lead to formation of indole **37**. Product **37** undergoes further degradation pathways, breaking down into aniline and aromatic hydrocarbons. Reactive intermediates in the system experience cleavage and decarbonylation processes, producing three key product streams: olefins, oxygen-containing compounds, and N-containing species, which ultimately evolve into pyridine derivatives and polycyclic aromatic frameworks (Fig. 3b, Route 5).

Graham demonstrated a multistep protocol for synthesizing furan-substituted oxazole derivatives from the reaction of furfural and methyl serine ester (Fig. 3b, Route 6) [40]. The synthetic route proceeds via several steps. First, the condensation of furfural with methyl serine ester in N, N-dimethylacetamide (DMA) solvent using K_2_CO_3_ and MgSO_4_ at room temperature yields oxazolidine **38**, then oxidation of **38** generates oxazole intermediate **39**. Subsequent isomerization and oxidation steps ultimately afford target oxazole product **40**. This methodology establishes a mild strategy for synthesizing bio-based oxazole compounds. Besides the above system, the Yan group used Pd@S-1 and H-beta as catalysts and carried out a series of decarbonylation and amination reactions at 460°C, 12 mL/min NH_3_ and 20 mL/min H_2_ to obtain a pyrrole skeleton in 75% yield (Fig. 3b, Route 7) [43]. Among them, the Pd@S-1 catalyst primarily catalyzes the decarbonylation of furfural to furan, the H-beta catalyst predominantly catalyzes the amination of furan to pyrrole, and the two catalysts successively facilitate the formation of pyrrole from furfural. Since the decarbonylation temperature of furfural is lower than that of amination, Pd@S-1 and H-beta catalysts should be placed in different areas of the fixed-bed reactor. Notably, this methodology can also successfully prepare D-proline starting from furfural through decarbonylation-amination, carboxylation, hydrogenation, and kinetic resolution.

### Levulinic acid

**Figure 4.**
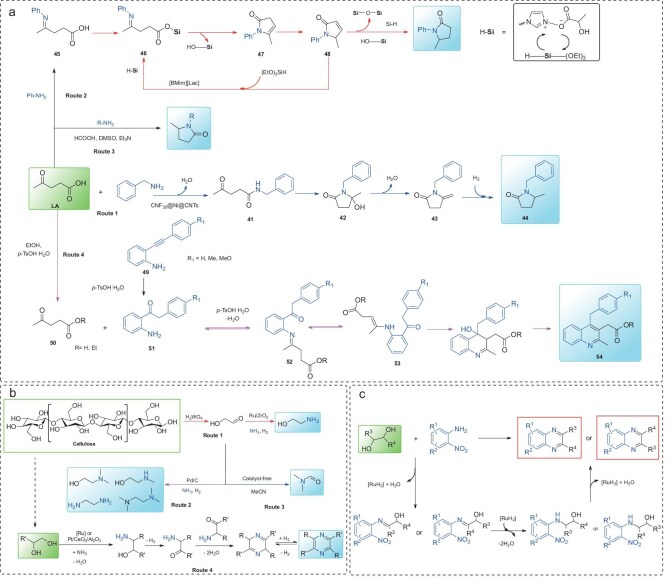
Reaction pathways for the synthesis of N-containing compounds from (a) LA and (b) cellulose and 1,2-diol conversion; (c) possible pathway for the formation of quinoxalines.

As an important C5 biomass platform compound, levulinic acid (LA) can be obtained by acid-catalyzed hydrolysis of C6 sugars [44]. Multiple synthetic approaches have been developed to transform LA into sustainable solvents, bio-based fuels, and key platform chemicals. Among them, much research has focused on developing and demonstrating methods to generate N-containing compounds, including pyrrolidone and quinoline derivatives. These products are widely applied in surfactants, organic solvents, pharmaceutical synthesis, and dyeing industries [4]. In detail, the direct conversion of LA to pyrrolidone proceeds via either an amination-reduction-cyclization pathway or an amination-cyclization-dehydrogenation-reduction pathway. Gao *et al.* [45] developed a novel catalyst composed of Ni nanoparticles stabilized by a porous carbon coating and supported on carbon nanotubes (CNF_x_@Ni@CNTs) for the reductive amination of LA with benzylamine. The CNF_30_@Ni@CNTs catalyst exhibited exceptional performance (99% yield of the product) with no significant deactivation observed even after 20 catalytic cycles. Characterization revealed that the porous carbon matrix conferred remarkable stability to CNF_30_@Ni@CNTs by effectively preventing Ni nanoparticles from leaching and sintering during reaction. Furthermore, the pathway is proposed in Fig. 4a, Route 1 based on the various characterizations and time-course reactions. This reaction initiates with the formation of amide intermediate **41**, followed by cyclization to intermediate **42**. Subsequent dehydration yields compound **43**, which undergoes hydrogenation to furnish the target product, 1-benzyl-5-methyl-2 -pyrrolidinone (**44**). Furthermore, a metal-free catalytic reductive amination of LA to pyrrolidones has been achieved in lactate-based ionic liquids, using triethoxysilane ((EtO)_3_SiH) as a reductant (Fig. 4a, Route 2) [46]. This provides a sustainable route for lactam synthesis under mild conditions. At 80°C, a series of lactams were obtained within 1 h with a yield of up to 96%, demonstrating catalytic efficiency. The proposed reaction pathway is illustrated. First, a condensation reaction between the benzylic amines and levulinic acid affords the ketimine intermediate **45**. This ketimine then reacts rapidly with triethoxysilane ((EtO)_3_SiH), which is pre-activated by the lactate-based ionic liquid [BMIm][Lac], yielding the silyl ether derivative **46**. Subsequently, the silyl ether undergoes intramolecular cyclization to give the cyclized intermediates **47** and/or **48**. Finally, under [BMIm][Lac] catalysis, these intermediates (**47**/**48**) are reduced by (EtO)_3_SiH to form the target N-alkyl-5-methyl-2-pyrrolidones. Throughout this process, [BMIm][Lac] acts as a multifunctional catalyst: it activates the Si-H bond of (EtO)_3_SiH to enhance its reducing ability, while simultaneously promoting the cyclization of the reaction intermediates. In addition, another example of catalyst-free synthesis of pyrrolidones from LA conversion is shown in Fig. 4a, Route 3, highlighting its simplicity, cost-effectiveness, and environmental sustainability. In this catalytic reaction, LA initially undergoes a condensation reaction with an amine in DMSO solvent, forming an iminium ion intermediate. Subsequently, formic acid acts as a hydride source, transferring a hydride ion to the iminium ion intermediate, which is rate-limiting for the overall reaction. The strongly basic nature of DMSO facilitates both the nucleophilic attack of the amine on the ketone and the nucleophilic attack of the formate ion on the iminium ion. This process serves as an extension of the classical Leuckart–Wallach reaction [47].

The novel approach toward the synthesis of quinoline derivatives from LA was developed by Ortiz-Cervantes *et al.* [48], as depicted in Fig. 4a, Route 4. The proposed mechanism proceeds as follows: alkyne hydration generates the corresponding ketone intermediate **51**. This ketone subsequently undergoes condensation with LA to form an imine intermediate **52** or **53**, which then undergoes sequential cyclization and dehydration-aromatization to yield the quinoline derivative **54**. Under the catalytic promotion of p-toluenesulfonic acid (PTSA), LA reacts with various 2-alkynylanilines through a cascade process: the Brønsted acid (PTSA) facilitates alkyne hydration to generate ketones, which subsequently participate in amine condensation. This is followed by cyclization and final dehydration-aromatization steps to construct substituted quinolines.

### Diols

Bio-derived vicinal diols (e.g. ethylene glycol, 1,2-propanediol, 2,3-butanediol, 1,2-pentanediol) can be efficiently produced from cellulose and hemicellulose. These diols serve as versatile feedstocks for value-added transformations into N-containing compounds, including ethanolamine, pyrazine derivatives, quinoxaline derivatives, and indole derivatives. As an example, Zhang *et al.* [49] reported a two-step approach for the synthesis of ethanolamine from cellulose. This process first involves the conversion of cellulose to glycolaldehyde, followed by the reductive amination of glycolaldehyde over a Ru-based catalytic system (Fig. 4b, Route 1). It is important to note that glycolaldehyde, which can be produced from glucose cracking in good yield, is regarded as a bio-based building block for the synthesis of N-containing compounds [50]. Apart from ethanolamine, Sels’ group described reductive amination of glycolaldehyde towards alkanolamines (Fig. 4b, Route 2) [51], and de Vries’ group developed catalyst-free selective N-formylation of secondary amines employing glycolaldehyde as the substrate (Fig. 4b, Route 3) [52].

Continued efforts are focused on developing an efficient conversion of bio-diols to pyrazines. A homogeneous acridine-based Ru-pincer complex enables acceptorless dehydrogenative coupling of 1,2-diols with ammonia, achieving exceptional pyrazine yields up to 99% under mild conditions [53]. Equally important, a heterogeneous Pt/CeO_2_/Al_2_O_3_ catalyst promotes a tandem process involving dehydrogenation, amination, coupling, and aromatization, yielding up to 94% pyrazines with enhanced reaction rates and recyclability [54]. Both systems operate via analogous mechanistic pathways: the hydroxyl group of the vicinal diol undergoes amination with ammonia under the action of the catalyst, forming a β-amino secondary alcohol intermediate. Subsequently, the secondary hydroxyl group in this intermediate undergoes dehydrogenation to be converted into an amino ketone intermediate. Then, two molecules of the amino ketone intermediate undergo self-coupling to form a six-membered ring precursor containing two nitrogen atoms; this precursor further undergoes two dehydration reactions to generate 2,5-dihydropyrazine. Finally, 2,5-dihydropyrazine undergoes dehydrogenative aromatization to yield the target pyrazine derivative (Fig. 4b, Route 4).

Quinoxaline derivatives have attracted significant attention in fluorescent probes, optoelectronic materials, and pharmaceutical intermediates [55]. The development of bio-quinoxaline production from biomass sources is of significant importance. Xie *et al.* [56] pioneered a hydrogen-transfer strategy for efficient quinoxaline synthesis from 2-nitroaniline and vicinal diols. Mechanistic studies revealed a Ru-catalyzed, base-mediated cascade reaction in which the initial dehydrogenation of the diol generates reactive carbonyl intermediates that subsequently undergo imination with 2-nitroaniline to form α-hydroxyimine species. Subsequent transfer hydrogenation of the nitro group, tautomerization, lactam cyclization, and dehydrogenative aromatization ultimately yield the desired quinoxaline scaffold (Fig. 4c). Notably, electron-donating substituents were found to significantly enhance reaction efficiency through electronic modulation.

## SYNTHESIS OF BIO-BASED N-CONTAINING COMPOUNDS FROM LIGNIN MODEL COMPOUNDS

Lignin is a three-dimensional network polymer primarily composed of three basic units: *p*-coumaryl alcohol, coniferyl alcohol, and sinapyl alcohol, which are interconnected via C–O and C–C bonds [3]. Most current research focuses on the selective cleavage of C–O/C–C bonds to produce valuable chemicals including phenols, arenes, aldehydes, ketones, acids, and cycloalkanes [3,57]. To further increase the value of lignin-derived products and broaden their applications, N-assisted depolymerization has gained considerable attention. Recent developments in nitrogen-induced depolymerization primarily involve lignin-derived monophenols, lignin dimer models, and realistic lignin as staring materials. This section reviews these strategies, emphasizing the pathways and mechanisms involved in C–O/C–C bond cleavage and C–N bond formation. Additionally, examples of pharmaceutical intermediates produced via these methods are discussed.

### Lignin phenolic monomers

**Figure 5.**
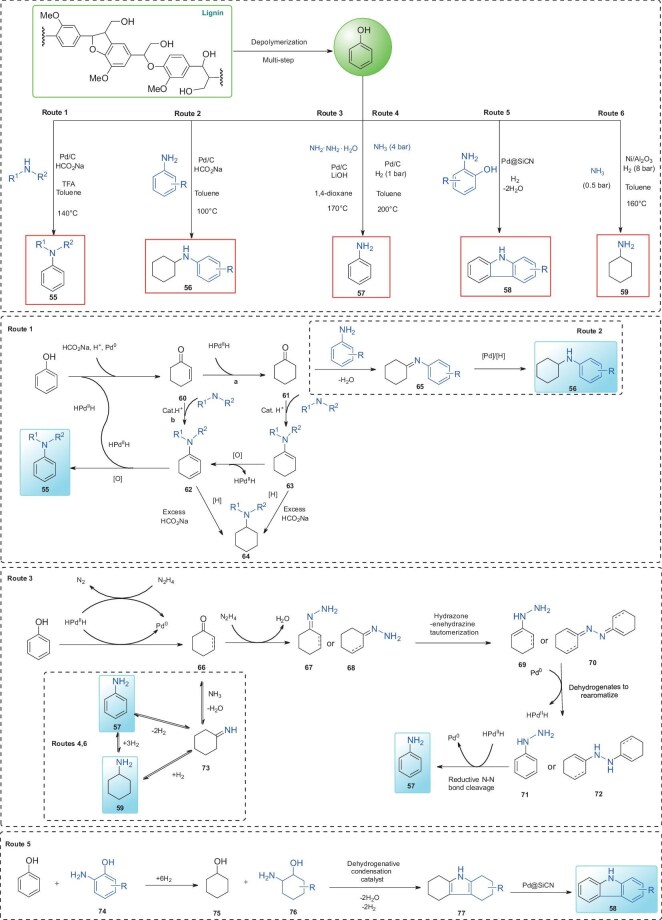
Various strategies using phenol as a substrate for the N-containing compound production.

Lignin monomers including (alkyl)phenols, (alkyl)syringol, and (alkyl)guaiacol can be obtained from lignin depolymerization via catalytic hydrogenation or oxidation reactions. Among these, phenol can be produced in high yields through dealkylation of these monomers or directly from lignin [58]. Due to its abundance and reactivity, phenol is commonly employed as a building block for synthesizing N-containing compounds in the presence of various nitrogen sources (Fig. 5). As an illustration, Li’s group developed three main routes utilizing aniline derivatives, alkylamines, and hydrazine hydrate as nitrogen sources, catalyzed by Pd/C [59,60]. In the first approach (Fig. 5, Route 1), direct cross-coupling of phenols with amines or anilines produce various substituted anilines. Lignin-derived monomers such as 4-propylphenol and 2-methoxyphenol are compatible with this system [59]. The proposed mechanism involves the formation of a palladium hydride species (HPd^II^H) from the reaction between HCO_2_Na and Pd/C. Phenol is initially reduced to intermediate **60**, which is further transformed into intermediate **61**. This species undergoes dehydration with an amine to yield compound **63**. In a parallel pathway, intermediate **60** can dehydrate to **62**, which then undergoes dehydrogenation to form the final product **55**. Additionally, in the presence of excess HCO_2_Na, hydrogenation of intermediates **62** and **63** leads to byproduct **64**. In a second route (Fig. 5, Route 2), using a similar catalytic system without trifluoroacetic acid (TFA) and with excess HCO_2_Na, the reaction predominantly yields a cyclohexylamine derivative (**56**). Here, intermediate **61** reacts with aniline derivatives through a dehydration step, followed by imine reduction to produce **56**. These results highlight the importance of reaction temperature and HCO_2_Na amount in determining the product distribution. Inorganic nitrogen sources, such as hydrazine hydrate and ammonia (NH_3_), can also be used as N-sources for N-containing chemical synthesis from lignin monomers. For example, using hydrazine hydrate as both a nitrogen and hydride source yields primary aniline (**57**) [60]. The proposed mechanism (Fig. 5, Route 3) involves HPd^II^H formation from hydrazine decomposition, and thereafter, phenol is reduced to cyclohexanone or compound **66**, which condenses with hydrazine to form hydrazone **67** or azine **68**. These intermediates undergo tautomerization to yield compounds **69** and **70**, which subsequently form phenylhydrazine derivatives **71** and **72** via dehydrogenation and rearomatization. Final N–N bond cleavage results in the formation of aniline (**57**). Under optimized conditions of 200°C, 4 bar NH_3_, and 1 bar H_2_ with a Pd/C catalyst a 98% yield of aniline was achieved (Fig. 5, Route 4) [60]. Interestingly, when 2-aminophenol (**74**) is used as the nitrogen source, the reaction pathway shifts significantly. Under the catalysis of Pd@SiCN, a nitrogen-heterocyclic compound, carbazole (**58**), is produced via a sequence of hydrogenation, dehydrogenation, and condensation reactions (Fig. 5, Route 5) [61]. This transformation highlights the potential of careful selection of nitrogen sources and catalysts to direct product selectivity and expand the diversity of N-containing aromatic compounds derived from lignin-based phenols. Overall, heterogeneous Pd-based catalysts (Pd/C, Pd@SiCN) are considered as efficient catalysts for the cross-coupling of phenols with amines, enabling the synthesis of N-containing aromatics under mild, functional-group-tolerant conditions. This efficiency arises from their high catalytic activity and selectivity, as well as their ability to facilitate two-electron processes crucial to the reaction mechanism. Besides Pd-based catalysts, Ni/Al_2_O_3_ has been shown to effectively catalyze over-hydrogenation of phenol intermediates, such as compound **73** or aniline (**57**), resulting in the formation of cyclohexane (**59**) in toluene (Fig. 5, Route 6) [62]. Interestingly, in these reactions, toluene is used as the best solvent for amination of phenol. Solvent choice significantly influences both the nucleophilicity and basicity of amines [9]. Yan’s group systematically investigated solvent effects using reductive amination of cyclohexanone as a model reaction over metal catalysts [63]. It is found that water is not a preferable solvent due to its preventing imine intermediate formation. Aprotic polar solvents have strong solvent–catalyst interactions that suppress hydrogenation activity and thereby reduce the rate of amine production. This is probably the main reason that no over-hydrogenation product cyclohexane is produced.

**Figure 6.**
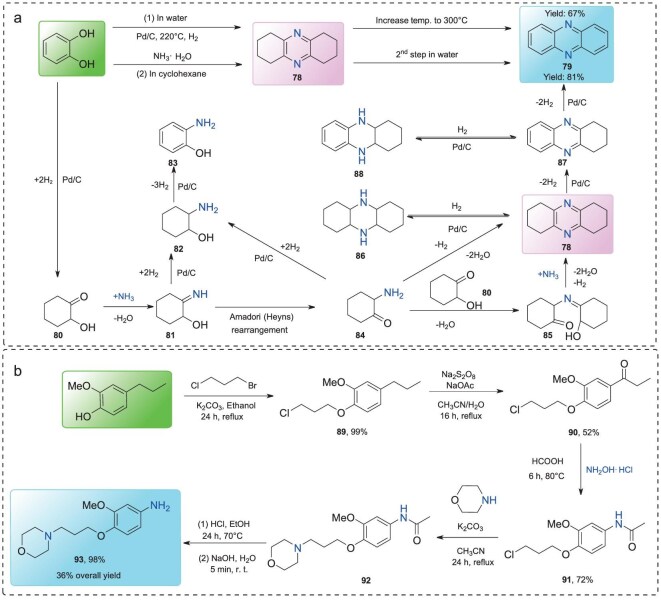
(a) The pathway of phenazine **79** synthesis from catechol; (b) synthesis of product **93** from lignin-derived 4-propylguaiacol.

Catechol, an important lignin-derived monomer, can be directly obtained via catalytic hydrogenolysis of C-lignin [64]. Yan’s group developed two catalytic systems for synthesizing phenazine (**79**) from catechol using a one-pot, two-step method in aqueous ammonia, catalyzed by Pd/C [65]. At 220°C, the reaction proceeds primarily through reductive amination (Fig. 6a), while at an elevated temperature of 300°C, dehydrogenation becomes the dominant pathway for forming the desired product, phenazine (**79**). The yield of **79** increases to 81% when a biphasic system of NH_3_·H_2_O and cyclohexane was employed. The proposed mechanism (Fig. 6a) begins with the hydrogenation of catechol, producing an intermediate that subsequently undergoes dehydration with ammonia to form compound **81**. This intermediate isomerizes into compound **84** via an Amadori or Heyns rearrangement, which rapidly reacts with intermediate **80** to generate compound **85** which is confirmed by GC-MS analysis. Subsequent amination of **85** with ammonia yields intermediate **78**, which then undergoes dehydrogenation over Pd/C to produce the final product, phenazine (**79**).

4-Propylguaiacol, another lignin-derived monomer, can be obtained in high yield via the hydrogenolysis of native lignin [66,67]. A novel approach has been developed to access valuable 3,4-dialkoxyanilines through a multistep transformation from 4-propylguaiacol (Fig. 6b) [68]. Using 1-bromo-3-chloropropane as the alkylating agent, 4-propylguaiacol is converted under basic conditions into compound **89**, which is then oxidized to **90**. A Beckmann rearrangement yields compound **91**, and subsequent substitution of the –Cl group by morpholine affords compound **92**. The final transformation into target product **93**, a key intermediate in the synthesis of various anticancer agents and agrochemicals, is accomplished in five steps, with an overall yield of 36%.

### Oxidized lignin β-O-4 model compounds

The β-O-4 linkage is the most prevalent, accounting for ∼50% of all linkages, depending on the lignin type. Due to a high bond dissociation energy (69.2 kcal·mol⁻¹) of the C_β_–O bond in phenolic β-O-4 units, direct cleavage is energetically demanding. However, oxidation of the C_α_–OH group in the β-O-4 motif to form a C_α_=O group significantly reduces the dissociation energy to 55.9 kcal·mol⁻¹, thereby facilitating bond cleavage [3,69]. As a result, oxidized β-O-4 model compound **94** is frequently employed as a representative substrate for further valorization studies. Chiba *et al.* explored a CuI-catalyzed transformation of oxidized β-O-4 compound **94** with alkyl secondary amines under oxygen atmosphere, resulting in the formation of amide derivatives **95** and **96** as major products (Table 1, Entry 1) [70]. This Cu-catalyzed oxidative amination offers a valuable route for the functionalization of lignin-derived substrates into N-containing fine chemicals. Building on the known pathways for the production of amides **95** and **96**, Liu’s group further developed a catalytic system for the synthesis of benzanilides (**97**) using aryl amines as nitrogen sources, with CuCl_2_ as the catalyst under air atmosphere (Table 1, Entry 2) [71]. Beyond organic amines, ammonia (NH_3_) can also react with oxidized β-O-4 model compound **94** in the presence of Cu(OAc)_2_, yielding both amides and α-ketoamides (Table 1, Entry 3) [72]. In this system, product selectivity is governed by the competitive interaction between ammonia and oxygen. Notably, Cu-based catalysts are essential for facilitating these transformations. To enhance atom economy and sustainability, a metal-free strategy for synthesizing benzanilides (**97**) from model compound **94** via oxidative bond cleavage has also been reported (Table 1, Entry 4) [73]. However, this approach is limited to specific amine sources—such as aniline—while others including morpholine, ammonium hydroxide, 2-aminopyridine, and NH_3_ fail to promote the reaction, likely due to structural incompatibilities during the oxidative process.

**Table 1.**
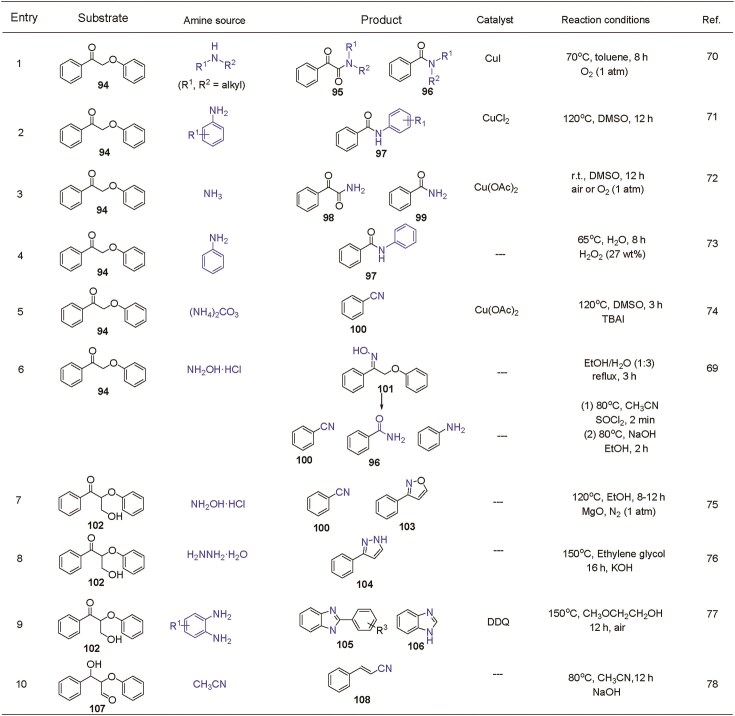
The production of N-containing compounds from oxidized lignin β-O-4 model compound.

In another notable development, benzonitrile (**100**) can be obtained from the transformation of oxidized β-O-4 compound **94** using (NH_4_)_2_CO_3_ as the nitrogen source, catalyzed by Cu(OAc)_2_ (Table 1, Entry 5) [74]. In this reaction, a hydrogen transfer from the C_β_–H to the C_α_ position occurs, triggering C_α_–C_β_ bond cleavage. This leads to the formation of a key benzaldehyde intermediate, which subsequently reacts with (NH_4_)_2_CO_3_ to afford benzonitrile **100**. Additionally, oxime **101** is formed via the reaction between compound **94** and hydrazine hydrate (H_2_NNH_2_·H_2_O) in an EtOH/H_2_O mixture (Table 1, Entry 6) [69]. Subsequent organic transformations of this oxime intermediate in a one-pot, two-step process yield a mixture of benzonitriles, benzamides, and anilines, achieving a total yield of up to 96% through a sequence of Beckmann rearrangement and hydrolysis.

Despite these advances, most systems exhibit low efficiency and selectivity when employing oxidized β-O-4 model compounds containing a γ-OH group as the starting material. To address this challenge, oxidized β-O-4 compound **102** with a γ-OH group has been efficiently converted into several valuable N-containing compounds (Table 1, Entries 7–10). For example, Wang’s group developed an NH_2_OH-mediated strategy to transform compound **102** into isoxazole and aromatic nitrile derivatives in the presence of MgO (Table 1, Entry 7) [75]. In this reaction, MgO facilitates both the oximation and intramolecular condensation steps. Mechanistic studies suggest that isoxazole product **103** is formed through condensation of the C_α_=O group with hydroxylamine, followed by a dehydration and cyclization process involving cleavage of the C_β_–O bond; whereas nitrile product **100** is formed via a Beckmann rearrangement or acidolysis pathway. Furthermore, a hydrazine-mediated cleavage of the oxidized β-O-4 motif containing a γ-OH group affords 3-arylpyrazole products (**104**) in a metal-free reaction (Table 1, Entry 8) [76]. In addition to these systems, organic amines have also been explored for depolymerization of such lignin-derived structures (Table 1, Entry 9). Interestingly, 2,3-dichloro-5,6-dicyano-1,4-benzoquinone (DDQ) acts as oxidant/catalyst for the conversion of β-O-4 dimer **102** to 2-phenylbenzimidazole derivatives (**105** and **106**), using o-phenylenediamines as the nitrogen source [77]. This catalytic system offers the advantage of utilizing both the C_β_ and C_γ_ atoms, minimizing by-product formation and achieving high atom economy. Oxidation of the C_γ_-OH group further facilitates C–O bond cleavage. Thus, selective oxidation of β-O-4 model compounds with C_γ_-OH to form compound **107** can be achieved using a combination of 2,2,6,6-tetramethylpiperidine N-oxide (TEMPO) and (diacetoxyiodo)benzene (BAIB). Using MeCN as both solvent and amine source, compound **107** is converted into cinnamonitriles (**108**) under basic conditions (Table 1, Entry 10) [78]. Mechanistic studies indicate that veratraldehyde is the key intermediate, formed via C_α_–C_β_ bond cleavage. Subsequent aldol condensation between veratraldehyde and nitriles leads to the formation of the desired cinnamonitrile products.

### Lignin β-O-4 model compounds

**Figure 7.**
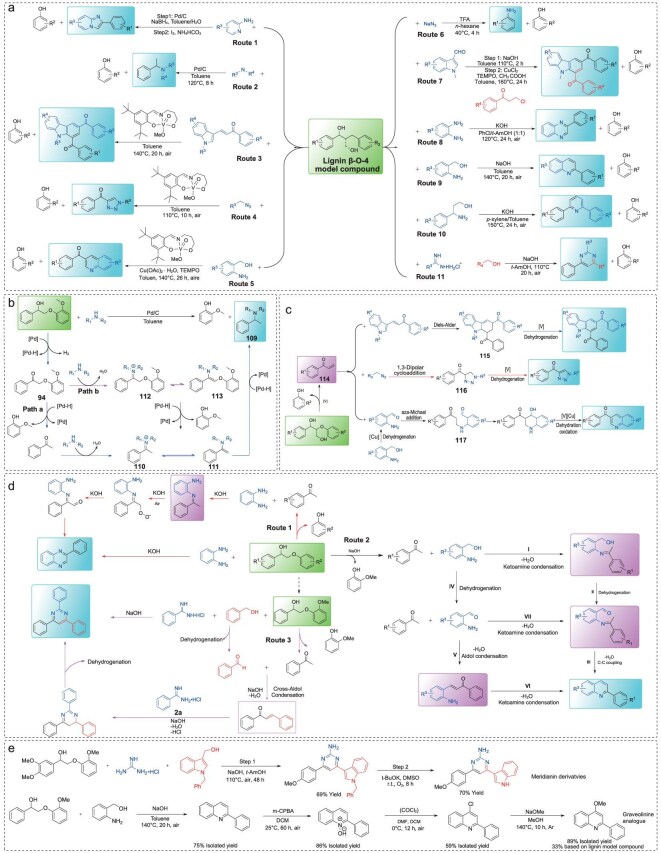
(a) Synthesis of N-containing aromatics from β-O-4 model compounds; and reaction pathways for the reductive amination of β-O-4 model compounds to (b) benzylamines; (c) N-heterocycles; (d) quinoxalines, quinolines and pyrimidines; (e) synthesis of meridianin derivative and graveolinine analogue.

The direct transformation of lignin β-O-4 model compounds into N-containing aromatics without a pretreatment step is highly desirable, yet remains a significant challenge. This is primarily because such processes require catalytic systems that are simultaneously compatible with C–O bond cleavage and C–N bond formation, while also suppressing competing reactions. Therefore, overcoming the barriers for direct conversion of these units is critical and provides a strategic foundation for producing N-containing aromatics from lignin-derived feedstocks. In this context, both the Jiao group and our group have developed a variety of efficient strategies utilizing different amine sources to synthesize N-heterocycles including imidazo[1,2-a]pyridines [79], benzylamines [80], carbazoles [81,82], triazoles [83], quinolines [84,85], anilines [86], quinoxalines [87], 2,6-diphenylpyridines [88], and pyrimidines [89] directly from β-O-4 model compounds, without requiring any pretreatment process (Fig. 7a).

The synthesis of N-containing aromatics from β-O-4 model compound transformations involves both transition-metal catalysis and base-promoted conversions. Among these, transition-metal catalysis offers advantages characterized by high catalytic activity for selective organic synthesis, abundant availability, and diverse chemical properties. For instance, Pd-based catalysts exhibit excellent catalytic activity for the transformation of phenol into N-containing aromatics. One-pot, two-step methods have been developed to synthesize imidazo[1,2-a]pyridines in yields up to 95% from phenolic lignin β-O-4 motifs in the presence of pyridine, I_2_, and Pd/C (Fig. 7a, Route 1) [79]. The first step selectively cleaves the C–O bond to form an acetophenone intermediate, followed by sp^3^ C–H bond oxidative activation and dehydration aromatization in the second step. Furthermore, we developed a one-pot Pd/C-catalyzed strategy for converting β-O-4 model compounds into benzylamines (Fig. 7a, Route 2) [80]. Notably, in this system secondary amines serve as both reducing agents and amine sources. This catalytic reaction exhibits broad functional group tolerance, affording a series of benzylamines in moderate to high yields. The transformation pathway involves two possible routes, proposed in Fig. 7b based on control experiments and time-course studies. The first pathway begins with dehydrogenation of phenolic β-O-4 model compounds to generate compound **94**, followed by C–O bond cleavage yielding acetophenone and guaiacol. Subsequently, amination produces intermediates **110** or **111**, which are hydrogenated to the desired product **109** (Fig. 7b, path a). Alternatively, compound **94** may react with amines to form imine **112** and its tautomer enamine **113**, which undergo C–O bond cleavage to release intermediates **110** or **111** (Fig. 7b, path b). However, these catalytic systems show low efficiency (yields below 40%) when applied to β-O-4 compounds containing a γ-OH group, due to a higher bond dissociation energy and steric hindrance. To address these challenges, the design of multifunctional catalysts that are highly efficient for β-O-4 structures with γ-OH groups and capable of promoting sequential reactions toward N-containing aromatics is critical.

Vanadium complexes coordinated with tridentate Schiff base ligands have been confirmed to exhibit high catalytic activity toward β-O-4 model compounds containing γ-OH groups [90]. Using V-based catalysts, sustainable strategies have been developed to synthesize carbazole [81], triazole [83], and quinoline [84] derivatives by appropriate nitrogen sources (Fig. 7a, Routes 3–5). In these reactions, an enone intermediate **114** plays a central role, undergoing cyclization and coupling addition with nitrogen sources such as indolenone, azides, and o-aminobenzaldehyde to yield the core scaffolds of target molecules **115–117** in Fig. 7c. These intermediates then undergo V-catalyzed dehydrogenation or dehydration to yield the desired products. The V-based catalyst acts as a bifunctional catalyst, facilitating both the selective C–O bond cleavage of the lignin model compounds and the subsequent dehydrogenation or dehydration steps.

Although transition-metal catalysts exhibit excellent performance in producing N-containing aromatics from direct conversion of phenolic β-O-4 model compounds, transition-metal-free approaches remain highly valuable owing to their simplicity and low cost. Jiao’s group demonstrated trifluoroacetic acid-mediated conversion of lignin model compounds to aniline derivatives in 40% yield using sodium azide as the amine source (Fig. 7a, Route 6) [86]. This finding may inspire further exploration of transition-metal-free synthesis of N-containing aromatics from lignin β-O-4 model compounds. Our group developed base-promoted one-pot cascade reactions for the preparation of N-containing aromatics including carbazoles [82], quinoxalines [87], quinolines [85], 2,6-diphenylpyridines [88] and pyrimidines [89], through direct conversion of β-O-4 model compounds without co-catalysts (Fig. 7a, Routes 7–11). Using the pathways to quinoxalines, quinolines, and pyrimidines as illustrative examples (Fig. 7d), mechanistic studies suggest that an excess of base promotes cleavage of the lignin model compound, facilitates dehydrogenation, and drives the coupling and cyclization reactions between acetophenone and amine sources to form key intermediates under basic conditions. Air is essential in this system by acting both as an oxidizing agent and a hydrogen acceptor during the dehydrogenation step. These strategies offer notable advantages over fossil fuel-based approaches, utilizing renewable substrates, avoiding transition-metal catalysts, operating under mild conditions, and eliminating the need for external hydrogen or oxidant agents, all within a straightforward one-pot process. Furthermore, the potential applications of these protocols are exemplified in the synthesis of pharmaceutical intermediates. For example, meridianin derivatives which display unique bioactivities and find applications in the pharmaceutical industry, and graveolinine analogues, which serve as precursors for various antibacterial, spasmolytic, and antitumor drugs, have been synthesized using these above methods (Fig. 7e) [85,89].

### Lignin 4-O-5 and α-O-4 model compounds

**Figure 8.**
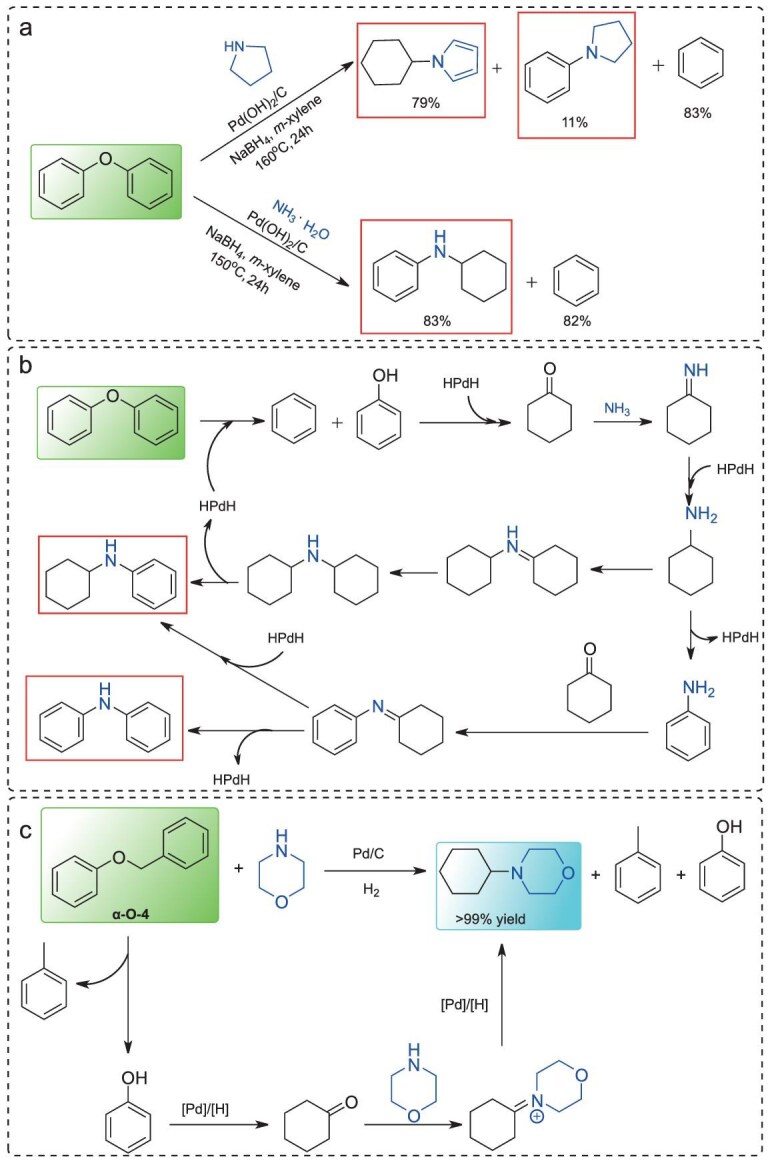
(a) N-induced polymerization of lignin 4-O-5 model compounds, (b) the pathway; (c) synthesis of 4-cyclohexylmorpholines from lignin α-O-4 model compounds.

Apart from lignin β-O-4 model compounds, other structural motifs containing C–O bonds include 4-O-5 and α-O-4 linkages, which account for ∼4% to 7% and 4% to 8% of total linkages, respectively, depending on the type of lignin. Among these ether bonds, the dissociation energy of the 4-O-5 bond is the highest (∼314 kJ/mol) [91], and it primarily appears in oligomer–oligomer couplings. A breakthrough was achieved by Li and co-workers, who developed a Pd(OH)_2_/C-catalyzed system for the direct conversion of 4-O-5 model compounds into aryl amines and cyclohexylamines in the presence of secondary amines, using NaBH_4_ as a reductant (Fig. 8a) [91]. Building on this, further progress was made by employing ammonia as the amine source in a similar catalytic system, affording aniline derivatives and arene products under mild conditions (Fig. 8a) [92]. Mechanistic studies indicate two pathways (Fig. 8b). First, HPdH species are generated from the reaction of Pd(OH)_2_/C with NaBH_4_ and water. This active species then facilitates the cleavage of the C–O bond in the 4-O-5 model compound, releasing phenol and benzene, which are subsequently reduced to cyclohexanone by HPdH. Cyclohexanone then reacts readily with ammonia to form an imine intermediate.

Lignin α-O-4 model compounds can be converted into 4-cyclohexylmorpholines using morpholine as the nitrogen source over Pd/C under H_2_ at 1 MPa, without any additives (Fig. 8c) [93]. Mechanistic studies reveal that the reaction begins with the Pd/C-catalyzed hydrogenolysis of the lignin α-O-4 model compound, releasing toluene and phenol. The phenol is then hydrogenated to cyclohexanone, which subsequently reacts with morpholine to form an imine intermediate. Finally, the imine undergoes hydrogenation to yield the target 4-cyclohexylmorpholine product.

## SYNTHESIS OF BIO-BASED N-CONTAINING COMPOUNDS FROM LIGNIN AND LIGNOCELLULOSE FEEDSTOCKS

**Figure 9.**
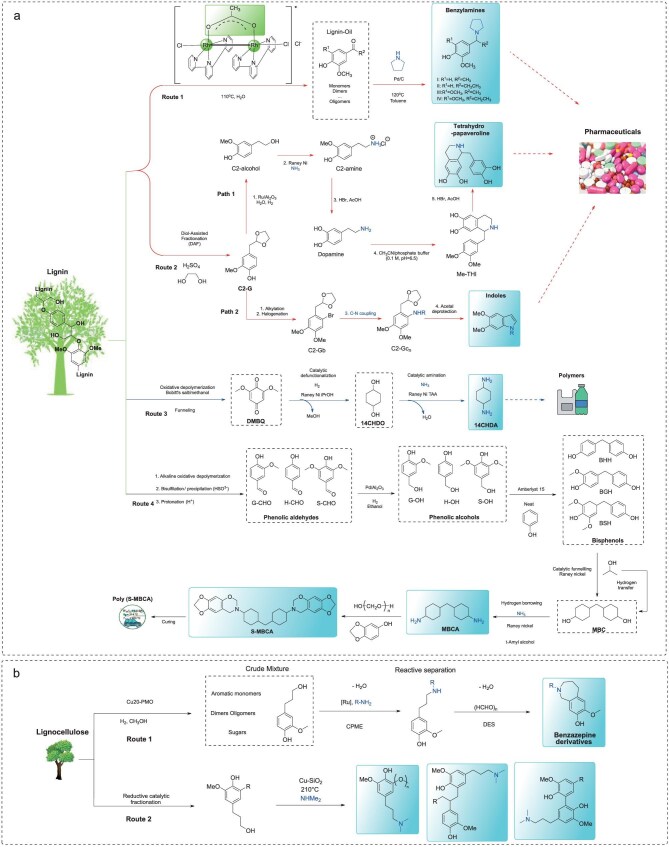
(a) The conversion of native lignin to N-containing compounds through multi-step methods; (b) synthesis of N-containing compounds from lignocellulose conversion.

Introducing nitrogen into the depolymerization of lignin to N-containing aromatics remains a significant challenge due to the intrinsic recalcitrance and structural complexity of lignin [3]. While many strategies have been developed for model compounds, their applicability to actual lignin feedstocks is not guaranteed because of this complexity. The most critical and difficult step in lignin depolymerization is the controlled cleavage of C–O and C–C bonds to generate the desired products, which then efficiently react with nitrogen sources. To date, direct conversion of lignin feedstocks into N-containing aromatics has been rarely reported. Existing useful strategies generally involve two or more steps within a one-pot reaction. That depolymerization of lignin gives lignin-derived platform chemicals—structurally similar to monolignols—as templates offers promising potential for designing synthetic pathways to produce both known and novel structures. The extraction of greater value from lignin is increasingly regarded as imperative for ensuring the economic sustainability of integrated biorefineries even though they use multi-step reactions. To achieve this, continuous efforts have been made. For example, our group developed a one-pot, two-step synthesis of benzylamines from organosolv lignin (Fig. 9a, Route 1) [78]. A lignin oil containing keto-functional groups is obtained over a Rh-based catalyst, which then reacts with pyrrolidine over Pd/C to yield benzylamines with a 0.4 wt% yield. This low yield is attributed to the dominance of phenolic aromatics and oligomers in the lignin oil, which pose major barriers to subsequent amination reactions. Moreover, delignification processing probably changes the lignin structure through the breakage of weak bonds, making lignin more stubborn, which also results in a lower yield of monomers. To functionalize the lignin products, Barta’s group has described several synthetic protocols for producing N-containing aromatics through multi-step reactions. In these strategies, depolymerization of lignin is used as the first step to release lignin monomers and oligomers, followed by the design of catalytic routes tailored to the specific product. For example, **C2-G** can be produced from softwood lignin in high yield through acidolysis and ethylene glycol stabilization. This compound serves as a platform molecule that offers biologically active compounds including tetrahydropapaveroline and indoles through multi-step reactions (Fig. 9a, Route 2) [94,95]. The same group further synthesizes 2,6-dimethoxybenzoquinone (DMBQ) and benzazepine derivatives. 1,4-Cyclohexanediamine (14CHDA), an important precursor for polyamide synthesis, can also be obtained from lignin through multi-step processing. In this route, DMBQ plays a crucial role. The following two steps—efficient defunctionalization of DMBQ to 1,4-cyclohexanediol (14CHDO) in 86.5% molar yield, and selective amination of 14CHDO with NH_3_ to produce 14CHDA in near-quantitative yield are both catalyzed by Raney^®^ Ni (Fig. 9a, Route 3) [96]. In addition, 4,4′-methylenebiscyclohexanamine (MBCA), a polymer building block, is synthesized in high yield via a sequence involving: oxidation–hydrogenation to obtain phenolic alcohols, electrophilic aromatic substitution using Amberlyst 15, demethoxylation/hydrogenation to methylenebiscyclohexanol (MBC) catalyzed by Raney^®^ Ni, and amination of MBC with NH_3_ through a hydrogen-borrowing strategy using lignosulfonate as the starting material (Fig. 9a, Route 4) [97].

Significant advances have been made in the transformation of lignocellulose into N-containing aromatics. Lignocellulose is composed of cellulose, hemicellulose, and lignin. Its conversion into N-containing aromatics presents a considerable challenge due to its complex structure and multi-component nature. Since lignin is the only component that inherently contains an aromatic structure, targeting the transformation of the lignin fraction in lignocellulose into N-containing aromatics is critical. Achieving this requires either the development of multifunctional catalysts or the adoption of a lignin-first strategy to circumvent inefficient or unprecedented pathways. Copper-doped porous metal oxides (Cu_2_O-PMO) have been developed for the transformation of lignocellulose into valuable chemicals [98]. Specifically, lignin is converted into aromatic monomers, primarily dihydroconiferyl alcohol, with smaller amounts of 4-ethylguaiacol and 4-propylguaiacol, while cellulose-rich solid residues are transformed into small aliphatic molecules, allowing for catalyst recovery and reuse. The same group demonstrated that the lignin monomer 4-(3-hydroxypropyl)-2-methoxyphenol serves as an ideal substrate for amine production [99]. A two-step reaction system was designed: Ru-catalyzed amination of 4-(3-hydroxypropyl)-2-methoxyphenol via a borrowing hydrogen strategy produced an amine-containing compound, which then underwent Pictet–Spengler cyclization in deep eutectic solvents (DESs) to afford benzazepine derivatives (Fig. 9b, Route 1). Similarly, Sels’ group also developed an approach for the synthesis of tertiary dimethylamines through a Cu-catalyzed hydrogen borrowing strategy (Fig. 9b, Route 2) [100]. These selected examples demonstrate promising strategies to enhance the economic feasibility of lignocellulosic biorefineries and provide sustainable synthetic routes for the pharmaceutical industry.

## CONCLUSIONS AND OUTLOOK

Biomass conversion is one of the most impactful areas of research for the production of value-added chemicals and fuels. Catalytic tools and organic synthesis methodologies have been extensively applied to enhance product diversity and facilitate chemical transformations. Among the key advances, the introduction of nitrogen functionality during lignocellulose degradation to N-containing compounds not only functionalizes the resulting products but also broadens their application potential.

This review has summarized recent progress in this area, focusing on the production of N-containing chemicals from platform molecules, lignin model compounds, lignin, and lignocellulose feedstocks. A chemocatalytic approach using biomass-derived intermediates—owing to their inherent chemical functionality and reactivity—offers promising potential for designing novel synthetic pathways. This strategy facilitates access to both known and novel compounds, thereby supporting the discovery and optimization of biologically active molecules inspired by natural products. Based on a comprehensive review of published studies, we have categorized and summarized the reaction pathways involved in cascade processes to assist researchers in selecting intermediates and designing effective coupling reactions in biomass valorization. Despite notable advancements, the development of this field still faces multiple challenges.

1. Developing multifunctional catalysts to reduce reaction steps: current approaches for nitrogen incorporation in lignocellulose conversion largely rely on platform molecules or lignin model compounds in one-pot reactions. However, when applied to lignin or lignocellulose, multi-step reactions are usually required. Reducing the number of steps not only lowers energy consumption but also enhances economic feasibility. Achieving this goal requires addressing structural complexity, heterogeneity, side reactions, and incompatible reaction conditions. Rational design and precise construction of multifunctional catalysts can modulate reaction pathways and enable efficient catalytic systems for the targeted synthesis of N-containing compounds with high yields and selectivity. Furthermore, advanced analytical methods combined with theoretical calculations are essential to uncover the underlying catalytic mechanisms, including C–O/C–C bond cleavage and C–N bond formation. A better understanding of these processes will improve catalytic efficiency and guide product design in future biomass transformations. The introduction of *in situ* external fields including force, electric, magnetic, light, and thermal fields is a promising approach to improve catalytic efficiency. Applying external fields enhances catalysis in energy and environmental applications through multiple mechanisms including optimized mass and energy transfer, dynamic modification of catalyst structures, and accelerated reaction kinetics, resulting in significantly improved catalytic performance. Moreover, advances in artificial intelligence (AI) are progressively transforming the field of scientific research. By integrating experimental studies with a variety of AI techniques—such as machine learning, deep learning, interpretability analysis, and coupled optimization algorithms—AI is being deeply embedded into biomass conversion processes. This integration supports the creation of innovative and efficient strategies for the targeted synthesis of N-containing compounds.
2. Separation and purification of N-containing compounds: isolating N-containing products is important for their use in pharmaceuticals, materials, and related industries. However, low selectivity and the broad distribution of products remain major barriers. Conventional column chromatography methods are not practical for large-scale industrial applications. Thus, integrated separation and purification techniques such as supercritical fluid extraction, high-speed countercurrent chromatography, membrane separation, microwave-assisted extraction, ultrasound-assisted extraction, and molecular distillation are potential solutions for achieving high-purity products.
3. Enhancing the diversity of biomass-derived products: current research in biomass valorization has mainly focused on value-added chemicals containing only C, H, and O atoms. In contrast, many high-value specialty chemicals and pharmaceuticals incorporate nitrogen, sulfur, phosphorus, or boron atoms. Although notable advances have been made in N-containing compound synthesis from lignocellulose, reports on S-, B-, or P-functionalized products remain scarce. Continued efforts in this direction may unlock new and scalable synthetic routes, significantly expanding the scope and impact of lignocellulose-based biorefineries.

## References

[1] Deng W, Feng Y, Fu J et al. Catalytic conversion of lignocellulosic biomass into chemicals and fuels. Green Energy Environ 2023; 8: 10–114. 10.1016/j.gee.2022.07.003

[2] Aziz T, Farid A, Haq F et al. A review on the modification of cellulose and its applications. Polymers 2022; 14: 3206. 10.3390/polym14153206 35956720 PMC9371096

[3] Reinhold JS, Pang J, Zhang B et al. Rhenium-based catalysts for biomass conversion Green Chem 2024; 26: 10661–86. 10.1039/D4GC02925A

[4] Murugesan K, Senthamarai T, Chandrashekhar VG et al. Catalytic reductive aminations using molecular hydrogen for synthesis of different kinds of amines. Chem Soc Rev 2020; 49: 6273–328. 10.1039/C9CS00286C 32729851

[5] Pelckmans M, Renders T, Van de Vyver S et al. Bio-based amines through sustainable heterogeneous catalysis. Green Chem 2017; 19: 5303–31. 10.1039/C7GC02299A

[6] Guillena G, Ramón DJ, Yus M. Hydrogen autotransfer in the N-alkylation of amines and related compounds using alcohols and amines as electrophiles. Chem Rev 2010; 110: 1611–41. 10.1021/cr9002159 19928825

[7] Müller TE, Hultzsch KC, Yus M et al. Hydroamination: direct addition of amines to alkenes and alkynes. Chem Rev 2008; 108: 3795–892. 10.1021/cr0306788 18729420

[8] Li H, Bunrit A, Li N et al. Heteroatom-participated lignin cleavage to functionalized aromatics. Chem Soc Rev 2020; 49: 3748–63. 10.1039/D0CS00078G 32458909

[9] Froidevaux V, Negrell C, Caillol S et al. Biobased amines: from synthesis to polymers; present and future. Chem Rev 2016; 116: 14181–224. 10.1021/acs.chemrev.6b00486 27809503

[10] Mika LT, Cséfalvay E, Németh Á . Catalytic conversion of carbohydrates to initial platform chemicals: chemistry and sustainability. Chem Rev 2018; 118: 505–613. 10.1021/acs.chemrev.7b00395 29155579

[11] Pingen D, Schwaderer JB, Walter J et al. Diamines for polymer materials via direct amination of lipid- and lignocellulose-based alcohols with NH_3_. ChemCatChem 2018; 10: 3027–33. 10.1002/cctc.201800365

[12] Qi H, Liu F, Zhang L et al. Modulating trans-imination and hydrogenation towards the highly selective production of primary diamines from dialdehydes. Green Chem 2020; 22: 6897–901. 10.1039/D0GC02280B

[13] Adhikary ND, Kwon S, Chung W-J et al. One-pot conversion of carbohydrates into pyrrole-2-carbaldehydes as sustainable platform chemicals. J Org Chem 2015; 80: 7693–701. 10.1021/acs.joc.5b01349 26176657

[14] Abdel-Kariem SM, Ali TE. The reaction of phosphorus decasulfide with some hydrazides and their hydrazones: new route for construction of four-membered, five-membered, and six-membered phosphorus heterocycles. J Heterocyclic Chem 2017; 54: 2916–21. 10.1002/jhet.2902

[15] Brust A, Cuny E. Conversion of reducing carbohydrates into hydrophilic substituted imidazoles. Green Chem 2013; 15: 2993–8. 10.1039/c3gc41203b

[16] EI Khadem H, Mohammed-Aly MM. 943. New anhydrophenylosazones. J Chem Soc 1963: 4929–32. 10.1039/JR9630004929

[17] El Khadem H, El-Shafei ZM, Abdel Rahman MMA. Acylated osazones and anhydro-derivatives. Carbohydr Res 1965; 1: 31–7. 10.1016/S0008-6215(00)80210-4

[18] El Ashry H, Atta KF, Aboul-Ela S et al. MAOS of sugar phenylosazones and their derived pyrazoles and triazoles. J Carbohydr Chem 2007; 26: 429–37. 10.1080/07328300701787164

[19] Chen X, Yang H, Hülsey MJ et al. One-step synthesis of N-heterocyclic compounds from carbohydrates over tungsten-based catalysts. ACS Sustain Chem Eng 2017; 5: 11096–104. 10.1021/acssuschemeng.7b03048

[20] Jia L, Makha M, Du C-X et al. Direct hydroxyethylation of amines by carbohydrates via ruthenium catalysis. Green Chem 2019; 21: 3127–32. 10.1039/C9GC01195A

[21] Caetano JAT, Fernandes AC. One-pot synthesis of amines from biomass resources catalyzed by HReO_4_. Green Chem 2018; 20: 2494–8. 10.1039/C8GC00915E

[22] Müller C, Diehl V, Lichtenthaler FW. Building blocks from sugars. Part 23. Hydrophilic 3-pyridinols from fructose and isomaltulose. Tetrahedron 1998; 54: 10703–12. 10.1016/S0040-4020(98)00634-6

[23] Hu L, Wu Z, Jiang Y et al. Recent advances in catalytic and autocatalytic production of biomass-derived 5-hydroxymethylfurfural. Renew Sustain Energy Rev 2020; 134: 110317. 10.1016/j.rser.2020.110317

[24] Guo R, Zeng Y, Lin L et al. CO_2_-assisted controllable synthesis of PdNi nanoalloys for highly selective hydrogenation of biomass-derived 5-hydroxymethylfurfural. Angew Chem Int Ed 2025; 64: e202418234. 10.1002/anie.202418234 PMC11796329 39434675

[25] Le N-T, Han Y, Lee K-I *et al.* Preparation of 2,5-bis(aminomethyl)furan by direct reductive amination of 2,5-diformylfuran over nickel-raney catalysts. Green Sustain Chem 2015; 5: 115. 10.4236/gsc.2015.53015

[26] Xu Z, Yan P, Xu W et al. Direct reductive amination of 5-hydroxymethylfurfural with primary/secondary amines via Ru-complex catalyzed hydrogenation. RSC Adv 2014; 4: 59083–87. 10.1039/C4RA10349A

[27] Wozniak B, Li Y, Hinze S et al. Efficient synthesis of biomass-derived N-substituted 2-hydroxymethyl-5-methyl-pyrroles in two steps from 5-hydroxymethylfurfural. Eur J Org Chem 2018; 2018: 2009–12. 10.1002/ejoc.201800171

[28] Zheng S, Wei Z, Wozniak B et al. Synthesis of valuable benzenoid aromatics from bioderived feedstock. Nat Sustain 2023; 6: 1436–45. 10.1038/s41893-023-01190-w

[29] Hidalgo FJ, Lavado-Tena CM, Zamora R. Conversion of 5-hydroxymethylfurfural into 6-(hydroxymethyl)pyridin-3-ol: a pathway for the formation of pyridin-3-ols in honey and model systems. J Agric Food Chem 2020; 68: 5448–54. 10.1021/acs.jafc.0c01679 32319769

[30] Larduinat M, Dokmak E, Verrier C et al. From 5-HMF to novel cyclopentenone-based aza spirocycles: an intramolecular aza-Piancatelli reaction in action. J Org Chem 2024; 89: 9661–5. 10.1021/acs.joc.4c00495 38888434

[31] Lei Z, Chen B, Koo Y-M et al. Introduction: ionic liquids. Chem Rev 2017; 117: 6633–5. 10.1021/acs.chemrev.7b00246 28535681

[32] Phan HB, Luong CM, Nguyen LP et al. Eco-friendly synthesis of 5-hydroxymethylfurfural and its applications as a starting material to synthesize valuable heterocyclic compounds. ACS Sustain Chem Eng 2022; 10: 8673–84. 10.1021/acssuschemeng.1c08211

[33] Mariscal R, Maireles-Torres P, Ojeda M et al. Furfural: a renewable and versatile platform molecule for the synthesis of chemicals and fuels. Energy Environ Sci 2016; 9: 1144–89. 10.1039/C5EE02666K

[34] Zhao K, Wen B, Tang Q et al. Recent catalytic innovations in furfural transformation. Green Chem 2024; 26: 9957–92. 10.1039/D4GC01983K

[35] Chen J, Li K, Huang Y et al. Research progress of chemical catalysis for biomass-based furfural to nitrogen-containing compounds. J Fuel Chem Technol 2024; 52: 1035–44. 10.19906/j.cnki.JFCT.2024007

[36] Zhang L, Ren Y, Liu W et al. Single-atom catalyst: a rising star for green synthesis of fine chemicals. Natl Sci Rev 2018; 5: 653–72. 10.1093/nsr/nwy077

[37] Qi H, Yang J, Liu F et al. Highly selective and robust single-atom catalyst Ru_1_/NC for reductive amination of aldehydes/ketones. Nat Commun 2021; 12: 3295. 10.1038/s41467-021-23429-w 34078894 PMC8172939

[38] Qi H, Li Y, Zhou Z et al. Synthesis of piperidines and pyridine from furfural over a surface single-atom alloy Ru_1_Co_NP_ catalyst. Nat Commun 2023; 14: 6329. 10.1038/s41467-023-42043-6 37816717 PMC10564752

[39] Mori A, Tanaka S, Ashida K et al. Preparation of fluorescent materials from biomass-derived furfural and natural amino acid cysteine through cross-coupling reactions for extended π-conjugation. Synlett 2015; 26: 1496–500. 10.1055/s-0034-1380460

[40] Graham TH . A direct synthesis of oxazoles from aldehydes. Org Lett 2010; 12: 3614–7. 10.1021/ol101346w 20704403

[41] Ferrer L, Mindt M, Wendisch VF et al. Indoles and the advances in their biotechnological production for industrial applications. Syst Microbiol and Biomanuf 2024; 4: 511–27. 10.1007/s43393-023-00223-x

[42] Yao Q, Xu L, Han Z et al. Production of indoles via thermo-catalytic conversion and ammonization of bio-derived furfural. Chem Eng J 2015; 280: 74–81. 10.1016/j.cej.2015.05.094

[43] Song S, Fung Kin Yuen V, Di L et al. Integrating biomass into the organonitrogen chemical supply chain: production of pyrrole and _D_-proline from furfural. Angew Chem Int Ed 2020; 59: 19846–50. 10.1002/anie.202006315 32720436

[44] Weingarten R, Conner WC, Huber GW. Production of levulinic acid from cellulose by hydrothermal decomposition combined with aqueous phase dehydration with a solid acid catalyst. Energy Environ Sci 2012; 5: 7559–74. 10.1039/c2ee21593d

[45] Gao G, Sun P, Li Y et al. Highly stable porous-carbon-coated Ni catalysts for the reductive amination of levulinic acid via an unconventional pathway. ACS Catal 2017; 7: 4927–35. 10.1021/acscatal.7b01786

[46] Wu C, Zhang H, Yu B et al. Lactate-based ionic liquid catalyzed reductive amination/cyclization of keto acids under mild conditions: a metal-free route to synthesize lactams. ACS Catal 2017; 7: 7772–6. 10.1021/acscatal.7b02231

[47] Wei Y, Wang C, Jiang X et al. Catalyst-free transformation of levulinic acid into pyrrolidinones with formic acid. Green Chem 2014; 16: 1093–6. 10.1039/C3GC42125B

[48] Ortiz-Cervantes C, Flores-Alamo M, García JJ. Synthesis of pyrrolidones and quinolines from the known biomass feedstock levulinic acid and amines. Tetrahedron Lett 2016; 57: 766–71. 10.1016/j.tetlet.2016.01.018

[49] Liang G, Wang A, Li L et al. Production of primary amines by reductive amination of biomass-derived aldehydes/ketones. Angew Chem Int Ed 2017; 56: 3050–4. 10.1002/anie.201610964 28156045

[50] Schandel CB, Høj M, Osmundsen CM et al. Thermaol cracking of sugars for the production of glycolaldehyde and other small oxygenates. ChemSusChem 2020; 13: 688–92. 10.1002/cssc.201902887 31849200

[51] Faveere W, Mihaylov T, Pelckmans M et al. Glycolaldehyde as a bio-based C_2_ platform chemical: catalytic reductive amination of vicinal hydroxyl aldehydes. ACS Catal 2020; 10: 391–404. 10.1021/acscatal.9b02437

[52] Flynn MT, Liu X, Dell’Acqua A et al. Glycolaldehyde as a bio-based C_1_ building block for selective *N*-formylation of secondary amines. ChemSusChem 2022; 15: e202201264. 10.1002/cssc.202201264 35947792 PMC9826180

[53] Daw P, Ben-David Y, Milstein D. Acceptorless dehydrogenative coupling using ammonia: direct synthesis of N-heteroaromatics from diols catalyzed by ruthenium. J Am Chem Soc 2018; 140: 11931–4. 10.1021/jacs.8b08385 30205675 PMC6502445

[54] Zhou Z, Guan W, Pan X et al. Synthesis of pyrazines from biomass-derived vicinal diols using ammonia over heterogeneous Pt/CeO_2_/Al_2_O_3_ catalysts. ACS Catal 2025; 15: 5664–73. 10.1021/acscatal.5c00372

[55] Soleymani M, Chegeni M. The chemistry and applications of the quinoxaline compounds. Curr Org Chem 2019; 23: 1789–827. 10.2174/1385272823666190926094348

[56] Xie F, Zhang M, Jiang H et al. Efficient synthesis of quinoxalines from 2-nitroanilines and vicinal diols via a ruthenium-catalyzed hydrogen transfer strategy. Green Chem 2015; 17: 279–84. 10.1039/C4GC01316F

[57] Li X, Ding Y, Pan X et al. Scission of C-O and C-C linkages in lignin over RuRe alloy catalyst. J Energy Chem 2022; 67: 492–9. 10.1016/j.jechem.2021.10.040

[58] Liao Y, Koelewijn S-F, Van den Bossche G et al. A sustainable wood biorefinery for low–carbon footprint chemicals production. Science 2020; 367: 1385–90. 10.1126/science.aau1567 32054697

[59] Chen Z, Zeng H, Girard SA et al. Formal direct cross-coupling of phenols with amines. Angew Chem Int Ed 2015; 127: 14695–9. 10.1002/ange.201506751 26531683

[60] Qiu Z, Lv L, Li J et al. Direct conversion of phenols into primary anilines with hydrazine catalyzed by palladium. Chem Sci 2019; 10: 4775–81. 10.1039/C9SC00595A 31160954 PMC6509994

[61] Forberg D, Schwob T, Kempe R. Catalytic condensation for the formation of polycyclic heteroaromatic compounds. Nat Commun 2018; 9: 1751. 10.1038/s41467-018-04143-6 29717125 PMC5931520

[62] Cuypers T, Morias T, Windels S et al. Ni-catalyzed reductive amination of phenols with ammonia or amines into cyclohexylamines. Green Chem 2020; 22: 1884–93. 10.1039/C9GC02625H

[63] Song S, Wang Y, Yan N. A remarkable solvent effect on reductive amination of ketones. Mol Catal 2018; 454: 87–93. 10.1016/j.mcat.2018.05.017

[64] Wang S, Zhang K, Li H et al. Selective hydrogenolysis of catechyl lignin into propenylcatechol over an atomically dispersed ruthenium catalyst. Nat Commun 2021; 12: 416. 10.1038/s41467-020-20684-1 33462206 PMC7814062

[65] Ren T, Qi W, He Z et al. One-pot production of phenazine from lignin-derived catechol. Green Chem 2021; 24: 1224–30. 10.1039/D1GC04102A

[66] Xiao L-P, Wang S, Li H et al. Catalytic hydrogenolysis of lignins into phenolic compounds over carbon nanotube supported molybdenum oxide. ACS Catal 2017; 7: 7535–42. 10.1021/acscatal.7b02563

[67] Wu Z, Bai H, Ji Y et al. Enhanced lignin depolymerisation to produce butylated hydroxytoluene and 4-propylguaiacol on carbon-nitride supported molybdenum catalysts. React Chem Eng 2023; 8: 1673–83. 10.1039/D3RE00115F

[68] Blondiaux E, Bomon J, Smoleń M et al. Bio-based aromatic amines from lignin-derived monomers. ACS Sustain Chem Eng 2019; 7: 6906–16. 10.1021/acssuschemeng.8b06467

[69] Wang Y, Du Y, He J et al. Transformation of lignin model compounds to *N*-substituted aromatics *via* Beckmann rearrangement. Green Chem 2018; 20: 3318–26. 10.1039/C8GC00920A

[70] Zhang J, Liu Y, Chiba S et al. Chemical conversion of β-O-4 lignin linkage models through Cu-catalyzed aerobic amide bond formation. Chem Commun 2013; 49: 11439–41. 10.1039/c3cc46912c 24169855

[71] Liu X, Zhang H, Wu C et al. Copper-catalyzed synthesis of benzanilides from lignin model substrates 2-phenoxyacetophenones under an air atmosphere. New J Chem 2017; 42: 1223–7. 10.1039/C7NJ02589K

[72] Li H, Liu M, Liu H et al. Amine-mediated bond cleavage in oxidized lignin models. ChemSusChem 2020; 13: 4660–5. 10.1002/cssc.202001228 32539209

[73] Liu X, Wang L, Zhai L et al. H_2_O_2_ -promoted C–C bond oxidative cleavage of β-O-4 lignin models to benzanilides using water as a solvent under metal-free conditions. Green Chem 2022; 24: 4395–8. 10.1039/D2GC00878E

[74] Luo Q, Tian S, Qiang Q et al. Copper-catalyzed C–C bond cleavage coupling with C≡N bond formation toward mild synthesis of lignin-based benzonitriles. J Environ Sci 2025; 151: 505–15. 10.1016/j.jes.2024.03.031 39481956

[75] Li H, Wang M, Liu H et al. NH_2_OH–mediated lignin conversion to isoxazole and nitrile. ACS Sustain Chem Eng 2018; 6: 3748–53. 10.1021/acssuschemeng.7b04114

[76] Hofmann LE, Hofmann D, Prusko L et al. Sequential cleavage of lignin systems by nitrogen monoxide and hydrazine. Adv Synth Catal 2020; 362: 1485–9. 10.1002/adsc.201901641

[77] Guo T, Liu T, He J et al. One-pot transformation of lignin and lignin model compounds into benzimidazoles. Eur J Org Chem 2022; 2022: e202101152. 10.1002/ejoc.202101152

[78] Qiang Q, Luo Q, Wang H et al. One-pot production of cinnamonitriles from lignin β-O-4 segments induced by selective oxidation of the γ-OH group. J Org Chem 2024; 89: 18424–35. 10.1021/acs.joc.4c02311 39655613

[79] Guo L, Ding Y, Wang H et al. Imidazo[1,2-a]pyridine derivatives synthesis from lignin β-O-4 segments via a one-pot multicomponent reaction. iScience 2023; 26: 106834. 10.1016/j.isci.2023.106834 37250767 PMC10209544

[80] Zhang B, Guo T, Liu Y et al. Sustainable production of benzylamines from lignin. Angew Chem Int Ed 2021; 60: 20666–71. 10.1002/anie.202105973 34297874

[81] Guo T, Lin Y, Pan D et al. Towards bioresource-based aggregation-induced emission luminogens from lignin β-O-4 motifs as renewable resources. Nat Commun 2023; 14: 6076. 10.1038/s41467-023-41681-0 37770462 PMC10539282

[82] Ji J, Ding C, Li S et al. Sustainable production of carbazole-based BioAIEgens from lignin major motifs. Green Chem 2024; 26: 3479–87. 10.1039/D3GC04384C

[83] Zhu W, Shi Y, Lu J et al. Sustainable production of triazoles from lignin major motifs. ChemSusChem 2024; 17: e202301421. 10.1002/cssc.202301421 38102854

[84] Zhu W, Skagfjörd Reinhold J, Lu J et al. Highly efficient transformation of lignin major segments into quinolines. Chem Eng Sci 2024; 290: 119899. 10.1016/j.ces.2024.119899

[85] Ding Y, Guo T, Li Z et al. Transition-metal-free synthesis of functionalized quinolines by direct conversion of β-O-4 model compounds. Angew Chem Int Ed 2022; 61: e202206284. 10.1002/anie.202206284 35869027

[86] Liu J, Qiu X, Huang X et al. From alkylarenes to anilines via site-directed carbon–carbon amination. Nat Chem 2019; 11: 94. 10.1038/s41557-018-0184-7 30410024

[87] Liu Y, Luo Q, Qiang Q et al. Successive cleavage and reconstruction of lignin β-O-4 models and polymer to access quinoxalines. ChemSusChem 2022; 15: e202201401. 10.1002/cssc.202201401 36055966

[88] Luo Q, Tian S, Qiang Q et al. Refining lignin into bioactive 2,6-diphenylpyridines via amine-participated [4 + 2] condensation strategy. Ind Crops Prod 2025; 226: 120631. 10.1016/j.indcrop.2025.120631

[89] Zhang B, Guo T, Li Z et al. Transition-metal-free synthesis of pyrimidines from lignin β-O-4 segments via a one-pot multi-component reaction. Nat Commun 2022; 13: 3365. 10.1038/s41467-022-30815-5 35690613 PMC9188570

[90] Hanson SK, Wu R, Silks LAP. C-C or C-O bond cleavage in a phenolic lignin model compound: selectivity depends on vanadium catalyst. Angew Chem Int Ed 2012; 51: 3410–3. 10.1002/anie.201107020 22266711

[91] Zeng H, Cao D, Qiu Z et al. Palladium-catalyzed formal cross-coupling of diaryl ethers with amines: slicing the 4-O-5 linkage in lignin models. Angew Chem Int Ed 2018; 57: 3752–7. 10.1002/anie.201712211 29384588

[92] Cao D, Zeng H, Li C-J. Formal cross-coupling of diaryl ethers with ammonia by dual C(Ar)–O bond cleavages. ACS Catal 2018; 8: 8873–8. 10.1021/acscatal.8b02214

[93] Zheng B, Song J, Wu H et al. Palladium-catalyzed synthesis of 4-cyclohexylmorpholines from reductive coupling of aryl ethers and lignin model compounds with morpholines. Green Chem 2020; 23: 268–73. 10.1039/D0GC03188G

[94] Afanasenko AM, Wu X, De Santi A et al. Clean synthetic strategies to biologically active molecules from lignin: a green path to drug discovery. Angew Chem Int Ed 2024; 63: e202308131. 10.1002/anie.202308131 37840425

[95] Castillo-Garcia AA, Haupenthal J, Hirsch AKH et al. Modular synthetic routes to biologically active indoles from lignin. Green Chem 2025; 27: 7506–12. 10.1039/D5GC01003A 40510740 PMC12151140

[96] Wu X, De bruyn M, Hulan JM et al. High yield production of 1,4-cyclohexanediol and 1,4-cyclohexanediamine from high molecular-weight lignin oil. Green Chem 2023; 25: 211–20. 10.1039/D2GC03777G 36685710 PMC9808896

[97] Wu X, Galkin MV, Barta K. A well-defined diamine from lignin depolymerization mixtures for constructing bio-based polybenzoxazines. Chem Catal 2021; 1: 1466–79. 10.1016/j.checat.2021.10.022

[98] Sun Z, Bottari G, Afanasenko A et al. Complete lignocellulose conversion with integrated catalyst recycling yielding valuable aromatics and fuels. Nat Catal 2018; 1: 82–92. 10.1038/s41929-017-0007-z

[99] Elangovan S, Afanasenko A, Haupenthal J et al. From wood to tetrahydro-2-benzazepines in three waste-free steps: modular synthesis of biologically active lignin-derived scaffolds. ACS Cent Sci 2019; 5: 1707–16. 10.1021/acscentsci.9b00781 31660439 PMC6813559

[100] Ruijten D, Narmon T, Van Aelst K et al. Tertiary amines from RCF lignin mono- and dimers: catalytic N-functionalized antioxidants from wood. ACS Sustain Chem Eng 2023; 11: 4776–88. 10.1021/acssuschemeng.2c07343

